# US Workers’ Self-Reported Mental Health Outcomes by Industry and Occupation

**DOI:** 10.1001/jamanetworkopen.2025.14212

**Published:** 2025-06-06

**Authors:** Aaron L. Sussell, Kristin Yeoman, Carol T. Nixon, Kenneth A. Scott, Tashina S. Robinson, Gerald S. Poplin

**Affiliations:** 1 Spokane Mining Research Division, National Institute for Occupational Safety and Health, Centers for Disease Control and Prevention, Spokane, Washington; 2 Western States Division, National Institute for Occupational Safety and Health, Centers for Disease Control and Prevention, Spokane, Washington

## Abstract

**Importance:**

Work-related hazards and stress have been shown to be associated with mental health, with suicide rates among adult workers increasing since 2000.

**Objective:**

To determine if self-reported lifetime diagnosed depression, frequent mental distress (FMD), extreme distress prevalences, and mean mentally unhealthy days (MUD) varied among current workers by industry or occupation.

**Design, Setting, And Participants:**

This cross-sectional study used Behavioral Risk Factor Surveillance System (BRFSS) data from 37 states reporting workers’ industry and occupation in 1 or more years between 2015 and 2019. The target population was currently employed civilian adults aged 18 years or older. Analyses were conducted in 2022 and 2023.

**Exposures:**

Workers’ current industry and occupation were the primary exposures of interest. Self-reported sociodemographic covariates included sex, age, race and ethnicity, education, coupled status, and health care coverage.

**Main Outcomes and Measures:**

Self-reported lifetime diagnosed depression, FMD, extreme distress, and MUD.

**Results:**

Of a total 536 279 workers assessed (unweighted sample, 535 997 workers; 263 007 female [49.1%]; 48 279 Hispanic [9.0%], 40 188 non-Hispanic Black [7.5%], 400 604 non-Hispanic White [74.7%]), 469 129 reported their industry or occupation. Lifetime diagnosed depression was reported by 80 319 of 534 342 workers (14.2% [95% CI, 13.9%-14.4%]). Mean MUD was 9.5 days (95% CI, 9.4-9.7 days) among 530 309 workers, and in all sociodemographic groups the mean MUD was 3 to 5 times higher among workers who reported lifetime diagnosed depression. Higher prevalences than all workers for lifetime diagnosed depression, FMD, and extreme distress were reported by workers who were female (lifetime diagnosed depression, 19.5% [95% 19.1%-19.9%]; FMD, 11.6% [95% CI, 11.3%-11.9%]; extreme distress, 4.8% [95% CI, 4.6%-5.1%]), ages 18 to 34 years (lifetime diagnosed depression, 16.9% [95% CI, 16.4%-17.3%]; FMD, 13.6% [95% CI, 13.1%-14.0%]; extreme distress, 5.5% [95% CI, 5.2%-5.8%]), and no longer or never in a couple (lifetime diagnosed depression, 18.0% [95% CI, 17.6%-18.4%]; FMD, 13.3% [95% CI, 12.9%-13.7%]; extreme distress, 5.7% [95% CI, 5.4%-6.0%]). By industry, retail trade (lifetime diagnosed depression: APR, 1.15 [95% CI, 1.05-1.25]; FMD: APR, 1.23 [95% CI, 1.10-1.39]) and accommodation and food services (lifetime diagnosed depression: APR, 1.13 [95% CI, 1.03-1.25]; FMD: APR, 6.8 [95% CI, 6.0-7.7]) had higher adjusted prevalences of lifetime diagnosed depression and FMD. By occupation, arts, design, entertainment, sports, and media (1.32 [95% CI, 1.09-1.60]); health care support (1.19 [95% CI, 1.03-1.38]); food preparation and serving (1.20 [95% CI, 1.05-1.36]); and sales and related occupations (1.13 [95% CI, 1.01-1.27]) had higher adjusted prevalences of FMD than the comparison group. Health care support (6.6% [95% CI, 5.5%-7.8%]), food preparation and service (6.9% [95% CI, 5.9%-7.8%]), building and grounds cleaning and maintenance (5.2% [95% CI, 4.4%-6.0%]), personal care and service (5.8% [95% CI, 4.9%-6.8%]), and sales and related occupations (4.8% [95% CI, 4.3%-5.3%]) had higher unadjusted extreme distress than all workers.

**Conclusions And Relevance:**

In this cross-sectional study, poor mental health among workers varied significantly by sociodemographic categories; significant differences among industry and occupation groups remained after adjustment. More research is needed on the effects of work-related factors on mental health, which may inform tailored treatment and prevention strategies.

## Key Points

**Question:**

Does mental health among adult workers vary by industry and occupation?

**Findings:**

This cross-sectional study used survey data collected in the years 2015 through 2019 from 536 279 civilian workers in 37 states. Among 469 129 workers who reported their industry and occupation, demographic information, and mental health outcomes, significant associations of industry and occupation with the prevalences of mental health outcomes were found.

**Meaning:**

These results suggest that workers' industry and occupation are associated with both positive and negative mental health outcomes.

## Teaser

This cross-sectional study of US workers examines the association of industry or occupation with depression, mental distress, and mentally unhealthy days.

## Introduction

There is evidence that psychosocial hazards at work, including work overload, shift work, long hours, lack of control over workload, social or physical isolation, poor communication, harassment, and interpersonal conflict are linked to workers’ poor mental health.^1,2,3,4,5,6,7^ Work-related injuries and illnesses may also adversely affect mental health. For example, Appalachian coal miners with severe work-related lung disease had more anxiety, depression, and suicidal ideation than did comparison groups.^8^ US suicide rates among adult workers have increased since the year 2000, with the highest rates in mining and construction industries.^9,10,11^

Depression is a common, episodic, serious mental illness that varies in severity and frequency, and symptoms often include a persistent feeling of sadness and loss of interest or pleasure in daily activities.^12^ It is associated with multiple risk factors including female sex, unmarried status, unemployment, chronic health conditions, and genomic and other biological factors.^12^ In 2020, nearly 1 in 10 of the US population aged 12 years or older experienced depression in the past 12 months, including 1 in 5 aged 18 to 25 years.^13^

The pattern of frequent mental distress (FMD) among all US adults (9%) is similar to that of depression: higher in women, the unemployed, people with disabilities, and groups with lower socioeconomic status.^14,15,16^ The estimated prevalence of extreme distress, an outcome defined as 30 mean mentally unhealthy days (MUD) in the past 30 days, increased from 3.6% to 6.4% in US adults in the years 1993 to 2019.^17^

In national analyses, adjusted FMD prevalence varied by nearly a factor of 3 among major occupation groups.^18^ Similarly, in Washington state (years 2006 and 2008), the prevalences of self-reported FMD and current depression diagnoses among workers varied significantly among occupation groups.^19^

This study was designed to measure workers’ mental health outcomes using a large representative sample. Our hypothesis was that there are significant differences in the prevalences of mental health outcomes among industry and occupation groups, the surrogates for work-related exposures. Another goal was to compare the prevalences of mental health outcomes in the mining industry with those in all other industries.

## Methods

### Data Source and Study Sample

The Behavioral Risk Factor Surveillance System (BRFSS) is an annual landline and cellphone random-selection survey of the US noninstitutionalized adult (ages 18 years and older) civilian population administered by the Centers for Disease Control and Prevention (CDC) and 50 states and 3 territories.^20^ The survey includes core and optional module questions. The CDC’s National Institute for Occupational Safety and Health (NIOSH) supports the industry and occupation optional module and assigns standardized industry and occupation classification codes from the US Census Bureau to participants’ free-text responses.^21,22^ NIOSH merged the industry and occupation module data with the public-use BRFSS data to create an industry and occupation dataset, which is not available to the public due the risk of inferential identity disclosure. BRFSS data (years 2015 to 2019) were aggregated to increase sample size using established procedures and became available in mid-2022.^21^ Analyses were completed in 2022 and 2023. Overall BRFSS response rates ranged from 45% (2017) to 49% (2019). The industry and occupation module was used by 37 states; participation by descending number of years was: 5 years (17 states), 4 years (3), 3 years (4), 2 years (7), and 1 year (6). The states’ unweighted sample size ranged from 1596 (Alaska) to 48 439 (Minnesota) respondents.

Due to the BRFSS survey design and weighting and raking, the weighted sample for each state is representative.^20,23,24^ In our dataset, 1 061 214 participants reported their employment status (11 174 [1%] refused). Of these, 536 279 were in the subpopulation of interest, civilian workers who reported their employment status was “employed for wages” or “self-employed.”

BRFSS questions elicited participants’ self-reported sociodemographic information, industry and occupation, and mental health outcomes.^25,26^ To prevent null values in small industry and occupation groups, we collapsed 20 BRFSS race and ethnicity categories to 5 using the codebook’s computed race variable (_RACE_G1), and collapsed “other race” and “multiracial” non-Hispanic categories, resulting in 4 categories: Hispanic, non-Hispanic Black, non-Hispanic White, and non-Hispanic other.

BRFSS was reviewed by the Human Research Protection Office of the CDC and determined to be exempt research. Survey information was collected under Office of Management and Budget. Results reporting in this study adhered to Strengthening the Reporting of Observational Studies in Epidemiology (STROBE) reporting guidelines.

### Exposures

Employed participants’ self-reported industry and occupation data were collected by trained interviewers and recorded as short, free-text entries. The occupation question was, “What kind of work do you do? For example, registered nurse, janitor, cashier, auto mechanic.” The industry question was, “What kind of business or industry do you work in? For example, hospital, elementary school, clothing manufacturing, restaurant.” NIOSH coded the free-text responses into US Census industry and occupation codes using the NIOSH Industry and Occupation Computerized Coding System and computer-assisted human coders.^27^ For analyses, the Census codes were grouped into major industry and occupation groups using the CDC National Health Interview Survey (NHIS) simple and detailed recodes in the Sample Adult Layout.^28^ Due to the authors’ research interest in the mining industry sector (simple recode 21), the mining sector was divided into its subsectors, mining except oil and gas extraction (hereafter referred to as *mining*) (detailed recode 07) and oil and gas extraction (OGE) (detailed recodes 06 and 08). Recode 08, “support activities for mining,” was grouped with OGE because we determined by keyword searching industry and occupation text responses for the mining industry that over 95% of the support activities workers are in OGE.

### Outcomes

Lifetime depression was defined as a positive response to the BRFSS question, “Has a doctor, nurse, or other health professional ever told you that you have a depressive disorder (including depression, major depression, dysthymia, or minor depression)?” MUD is the other primary outcome and 1 of 4 validated health-related quality of life (HRQOL-4) (healthy days) measures in the BRFSS.^17,18,29^ It is based on the numeric response (on a scale from 0 to 30) to the question, “Now thinking about your mental health, which includes stress, depression, and problems with emotions, for how many days during the past 30 days was your mental health not good?”^25^ MUD has high specificity and positive predictive value for reporting at least 4 depression symptoms on standardized questionnaires.^30^

Two secondary outcomes were calculated from MUD. FMD is defined as a MUD score of 14 or higher and extreme distress as a MUD score of 30, both in reference to the past 30 days. FMD has been widely used as a surrogate for depression symptoms in population health studies.^14,15,19,31,32^ Extreme distress has been reported once previously, and unlike the other measures, it has not been validated against standardized questionnaires.^17^

### Statistical Analysis

Because sociodemographic characteristics, health behaviors, and health care coverage were associated with both outcomes and exposures and thus were potentially confounders, we adjusted for the following covariates in multivariable logistic regression models: sex (male or female), age (18 to 34, 35 to 44, 45 to 54, 55 to 64, and 65 or more years); race and ethnicity (Hispanic, non-Hispanic Black, non-Hispanic White, and non-Hispanic other); educational attainment (less than high school graduate, high school graduate or GED, some college or technical school, and graduated from college or technical school), coupled status (married or in unmarried couple, no longer coupled [divorced, widowed, separated], or never in a couple), and health care coverage (yes or no).

SAS version 9.4 (SAS Institute Inc) and SAS-callable SUDAAN (RTI International) were used for analyses to account for the BRFSS complex survey design and sample weighting. Where necessary for the SUDAAN analyses, dichotomous outcome variables were recoded to (1 [yes], 0 [no]) and covariates to consecutive integers. Unless otherwise noted, estimates reported in this study (eg, counts, percentages, means, and 95% CIs) are weighted estimates for the 37 states over the 5-year period. In SAS and SUDAAN, the DOMAIN and SUBPOPX statements were used, respectively, to specify the subpopulation of interest (currently employed civilians). SAS Proc SURVEYFREQ and SURVEYMEANS were used for descriptive analyses with options VARMETHOD Taylor and NOMCAR. SUDAAN Proc RLOGIST was used to calculate weighted adjusted prevalence ratios (APRs) and their 95% CIs with respect to the reference industry and occupation groups. In the adjusted models, the reference groups for industry (public administration) and occupation (transportation and material moving) were selected based on their lifetime diagnosed depression prevalences being near the median among the respective groups. APRs were significant at the α = .05 level if the 95% CI did not include 1.0. For 2-way comparisons of groups’ unadjusted prevalences or means, the outcomes were considered to be significantly different if their 95% CIs did not overlap. Nonoverlapping CIs was chosen as a conservative definition of statistical significance, equivalent to a *P* value of <.01 when comparing the means for 2 groups of equal size and variability.^33^ For the unadjusted analyses, the reference group was all workers combined.

## Results

Among 536 279 eligible employed participants, 57 563 (10.7%) and 74 450 (13.9%) had missing or noncodable industry and occupation responses, respectively, and were excluded, resulting in 478 716 with industry and 461 929 with occupation responses.^21^ Percentage missing for other variables were: race and ethnicity 2.9% (30 120 participants); sex, age, education, coupled status, and health care coverage (range, 1.1%-1.7% [11 747-1 717 501 participants]); lifetime depression diagnosis 1.5% (15 923 participants), and mentally unhealthy days 2.8% (28 920 participants). Income was not used in this study because its non-random missingness of 17.6% (188 919 participants) was deemed unacceptable.

The prevalences of lifetime diagnosed depression, FMD, and extreme distress in all workers combined was 14.2% (95% CI, 13.9%-14.4%), 9.6% (95% CI, 9.4%-9.8%), and 4.1% (95% CI, 3.9%-4.2%), respectively (Table 1). The mean MUD for all workers with lifetime diagnosed depression was 9.5 days (95% CI, 9.4-9.7 days) and for those without it, 2.2 days (95% CI, 2.2-2.3 days). Across all sociodemographic groups, there was significant variability in the prevalence of extreme distress (range, 2.2%-6.3%). Across sociodemographic groups, mean MUD were 3 to 5 times higher among workers who reported lifetime diagnosed depression (range, 6.1-11.7 days) than in those who did not (range, 1.1-3.1 days). Sociodemographic groups with the highest mean MUD, among workers with lifetime diagnosed depression, were age 18 to 34 years (11.3 [95% CI, 11.0-11.7]), no longer or never in a couple (11.0 [95% CI, 10.7-11.3]), and no health care coverage (11.9 [95% CI, 11.3-12.6]); these groups also had the highest mean MUD among workers without lifetime depression diagnosis (Table 1). Lifetime diagnosed depression, FMD, and extreme distress prevalences were inversely associated with age. The prevalence of lifetime diagnosed depression in female participants (19.5% [95% CI, 19.1%-19.9%]) was double that for male participants (9.8% [95% CI, 9.8%-10.1%]). Female participants in the groups aged 18 to 34 years and who were no longer or never in a couple had higher prevalences than all workers of lifetime diagnosed depression, FMD, and extreme distress. Workers with no health care coverage had higher prevalences of FMD (13.6% [95% CI, 12.8-14.4]), extreme distress (6.7% [95% CI, 6.0%-7.3%]), and mean MUD among those with lifetime depression diagnosis (11.9 [95% CI, 11.3-12.6]) than all workers, but not lifetime diagnosed depression. Non-Hispanic White participants (16.6% [95% CI, 16.3%-16.9%]) and participants with some college or technical school education (16.2% [95% CI, 15.7-16.7%]) had higher prevalences of lifetime diagnosed depression than all workers, but not FMD and extreme distress. Non-Hispanic workers who did not identify as White or Black had lower prevalences for all mental health outcomes except mean MUD.

**Table 1.** Workers’ Mental Health Outcomes by Sociodemographic Factors, Behavioral Risk Factor Surveillance System, 37 States, 2015-2019^a^

Sociodemographic factors	Sample No, unweighted	% (95% CI)			MUD by depression status, mean (95% CI)	
		Lifetime diagnosed depression	Frequent mental distress	Extreme distress	Yes	No
All workers combined	535 997	14.2 (13.9-14.4)	9.6 (9.4-9.8)	4.1 (3.9-4.2)	9.5 (9.4-9.7)	2.2 (2.2-2.3)
Sex						
Male	272 990	9.8 (9.5-10.1)	8.0 (7.7-8.3)	3.4 (3.2-3.6)	9.4 (9.1-9.7)	2.0 (1.9-2.0)^b^
Female	263 007	19.5 (19.1-19.9)^c^	11.6 (11.3-11.9)^c^	4.8 (4.6-5.1)^c^	9.6 (9.4-9.8)	2.5 (2.5-2.6)^c^
Age, y						
18-34	119 920	16.9 (16.4-17.3)^c^	13.6 (13.1-14.0)^c^	5.5 (5.2-5.8)^c^	11.3 (11.0-11.7)^c^	3.1 (3.0-3.2)^c^
35-44	97 934	14.0 (13.5-14.5)	9.3 (8.9-9.8)	3.7 (3.5-4.0)	9.1 (8.8-9.5)	2.2 (2.1-2.3)
45-54	124 543	12.6 (12.2-13.0)^b^	7.5 (7.2-7.8)^b^	3.4 (3.1-3.6)^b^	8.5 (8.1-8.8)^b^	1.8 (1.7-1.9)^b^
55-64	133 397	12.3 (11.9-12.8)^b^	6.6 (6.2-6.9)^b^	3.1 (2.8-3.3)^b^	7.6 (7.2-8.0)^b^	1.5 (1.4-1.6)^b^
≥65	60 485	10.0 (9.3-10.7)^b^	4.6 (4.0-5.2)^b^	2.2 (1.9-2.5)^b^	6.1 (5.5-6.8)^b^	1.1 (1.0-1.2)^b^
Race and ethnicity						
Hispanic	48 279	11.1 (10.4-11.7)^b^	9.8 (9.1-10.5)	4.3 (3.8-4.8)	10.5 (9.8-11.2)^c^	2.3 (2.2-2.5)
Non-Hispanic Black	40 188	10.5 (9.8-11.2)^b^	10.3 (9.6-10.9)	4.6 (4.2-5.1)	10.9 (10.2-11.6)^c^	2.5 (2.3-2.6)
Non-Hispanic White	400 604	16.6 (16.3-16.9)^c^	9.6 (9.4-9.9)	4.0 (3.8-4.1)	9.1 (8.9-9.3)^b^	2.1 (2.1-2.2)
Non-Hispanic other^d^	38 192	9.3 (8.6-10.1)^b^	8.1 (7,3-8.9)^b^	3.1 (2.7-3.5)^b^	10.2 (9.4-10.9)	2.0 (1.9-2.2)
Education						
Less than high school	25 882	13.7 (12.7-14.6)	12.2 (11.2-13.2)^c^	6.3 (5.6-7.0)^c^	11.7 (10.9-12.6)^c^	2.5 (2.2-2.7)
High school graduate or GED	124 578	13.2 (12.8-13.7)	11.1 (10.7-11.7)^c^	5.0 (4.7-5.3)^c^	10.9 (10.4-11.3)^c^	2.5 (2.4-2.6)^c^
Some college or technical school	146 419	16.2 (15.7-16.7)^c^	10.7 (10.3-11.1)^c^	4.5 (4.2-4.8)	9.9 (9.6-10.2)^c^	2.4 (2.3-2.4)
College graduate or more	237 965	13.2 (12.9-13.5)^b^	6.8 (6.6-7.1)^b^	2.4 (2.3-2.6)^b^	7.6 (7.3-7.8)^b^	1.9 (1.8-1.9)^b^
Coupled status						
Married or in unmarried couple	330 720	11.6 (11.4-11.9)^b^	7.1 (6.9-7.4)^b^	3.0 (2.8-3.1)^b^	8.0 (7.8-8.3)^b^	1.8 (1.8-1.9)^b^
No longer or never in a couple	202 195	18.0 (17.6-18.4)^c^	13.3 (12.9-13.7)^c^	5.7 (5.4-6.0)^c^	11.0 (10.7-11.3)^c^	2.9 (2.8-2.9)^c^
Health care coverage						
Yes	483 508	14.1 (13.9-14.4)	9.0 (8.8-9.2)^b^	3.7 (3.5-3.8)^b^	9.2 (9.0-9.4)	2.1 (2.1-2.2)
No	51 057	14.3 (13.6-15.1)	13.6 (12.8-14.4)^c^	6.7 (6.0-7.3)^c^	11.9 (11.3-12.6)^c^	3.0 (2.8-3.2)^c^

^a^ States contributing data were: Alaska, California, Colorado, Connecticut, Delaware, Florida, Georgia, Hawaii, Idaho, Illinois, Iowa, Kansas, Louisiana, Maryland, Massachusetts, Michigan, Minnesota, Mississippi, Missouri, Montana, Nebraska, New Hampshire, New Jersey, New Mexico, New York, North Carolina, North Dakota, Pennsylvania, Rhode Island, South Carolina, Tennessee, Texas, Utah, Vermont, Washington, West Virginia, Wisconsin.

^b^ Significantly lower than all workers combined.

^c^ Significantly higher than all workers combined.

^d^ Respondents selected other race or multiracial.

Mining and OGE had larger proportions of male workers (88% and 91%, respectively) than all other industries (50%), and smaller sample sizes, resulting in more imprecise and unreportable estimates for female workers. Male workers in mining had a significantly lower proportion of younger workers (ages 18 to 34 years: mining, 21.4% [95% CI, 15.3%-27.6%] vs all other industries, 33.0% [95% CI, 32.6%-33.5%]), and male and female workers in mining had a higher proportion of non-Hispanic White workers than all other industries (88.6% [95% CI, 83.0%-94.3%] vs 60.6% [95% CI, 60.1%-61.2%] and 83.4% [95% CI, 70.4%-96.4%] vs 61.3% [95% CI, 60.8%-61.9%], respectively) (Table 2). Male workers in mining and OGE had significantly lower proportions of college graduates (12.3% [95% CI, 8.5%-16.0%] and 20.0% [95% CI, 15.5%-24.5%], respectively) than all other industries (30.5% [95% CI, 30.1%-30.9%]). In mining, the prevalences of lifetime diagnosed depression among male workers (5.7% [95% CI, 3.2%-8.2%] vs 9.9% [95% CI, 9.6%-10.2%]) and of FMD among female workers (4.7% [95% CI, 0.3%-9.0%] vs 11.5% [95% CI, 11.2%-11.9%]) were significantly lower than in all other industries for the respective sex.

**Table 2.** Workers’ Sociodemographic Factors and Mental Health Outcomes by Sex for Mining Subsectors and All Other Industries, Behavioral Risk Factor Surveillance System, 37 States, 2015-2019^a^

Sociodemographic factors	Workers, % (95% CI)					
	Male			Female		
	Mining	Oil and gas extraction	All other industries	Mining	Oil and gas extraction	All other industries
Total, unweighted No. (weighted) [n = 498 351 (103 515 606)]	849 (149 787)	2464 (472 289)	248 581 (56 090,11)	120 (12 079)	252 (49 407)	246 085 (46 741 926)
% (95% CI)	0.26 (0.20-0.32)	0.83 (0.71-0.96)	98.9 (98.8-99.0)	0.03 (0.02-0.04)	0.10 (0.06-0.15)	99.9 (99.8-99.9)
Age, y						
18-34	21.4 (15.3-27.6)^b^	38.1 (30.9-45.2)	33.0 (32.6-33.5)	23.4 (9.7-37.3)	33.4 (12.9-54.0)^c^	30.8 (30.3-31.3)
35-44	22.7 (14.1-31.2)	26.6 (18.5-34.8)	22.2 (21.8-22.7)	35.7 (12.7-58.6)^c^	13.5 (4.4-22.6)^c^	21.8 (21.4-22.3)
45-54	23.6 (13.5-33.7)	15.0 (11.2-18.7)^b^	21.6 (21.2-22.0)	24.0 (8.1-39.9)^c^	39.6 (14.8-64.3)^c^	22.8 (22.4-23.2)
55-64	26.5 (14.5-38.4)	15.2 (11.1-19.3)	16.8 (16.5-17.2)	15.2 (6.2-24.3)^c^	12.0 (3.8-20.1)^c^	18.5 (18.1-18.9)
≥65	5.8 (0.9-10.7)^c^	5.1 (2.9-7.3)	6.3 (6.1-6.6)	NA^d^	1.5 (0.5-2.6)^c^	6.1 (5.9-6.3)
Race and ethnicity						
Hispanic	6.9 (1.8-12.1)^b^^,^^c^	26.8 (17.4-36.1)	20.3 (19.8-20.8)	NA^d^	NA^d^	16.7 (16.2-17.2)
Non-Hispanic Black	NA^d^	3.9 (2.6-5.2)^b^	10.1 (9.8-10.4)	NA^d^	NA^d^	13.2 (12.9-13.6)
Non-Hispanic White	88.6 (83.0-94.3)^e^	64.8 (56.2-73.5)	60.6 (60.1-61.2)	83.4 (70.4-96.4)^e^	79.3 (61.3-97.3)^e^	61.3 (60.8-61.9)
Non-Hispanic other^f^	2.7 (1.2-4.2)^b^	4.5 (2.4-6.7)^b^	9.0 (8.6-9.4)	4.5 (0.4-8.6)^c^	NA^d^	8.8 (8.3-9.2)
Education						
Less than high school	16.6 (5.6-27.6)^c^	19.2 (10.2-28.2)	12.3 (11.9-12.7)	NA^d^	NA^d^	7.7 (7.4-8.1)
High school graduate or GED	50.7 (39.5-61.9)	31.5 (25.4-37.6)	28.0 (27.5-28.4)	17.2 (4.1-30.3)^c^	38.1 (13.6-62.6)^c^	22.6 (22.1-23.0)
Some college or technical school	20.4 (14.4-26.4)	29.3 (23.2-35.4)	29.2 (28.8-29.7)	52.7 (33.0-72.4)	38.5 (17.0-60.1)	32.6 (32.1-33.1)
College graduate or more	12.3 (8.5-16.0)^b^	20.0 (15.5-24.5)^b^	30.5 (30.1-30.9)	25.9 (12.6-39.3)	21.3 (7.9-34.6)^b^^,^^c^	37.1 (36.6-37.6)
Coupled status						
Married or in unmarried couple	76.8 (70.1-83.6)^e^	71.6 (66.0-77.2)^e^	62.0 (61.5-62.5)	64.4 (46.9-82.0)	70.6 (54.8-86.4)	58.1 (57.6-58.6)
No longer or never in a couple	23.2 (16.4-29.9)^b^	28.4 (22.8-34.0)^b^	38.0 (37.5-38.5)	35.6 (18.0-53.1)	29.4 (13.6-45.2)	41.9 (41.4-42.4)
Health care coverage						
Yes	89.1 (78.7-99.4)	88.7 (83.9-93.6)	84.4 (84.0-84.8)	89.3 (74.7-100.0)	80.2 (53.5-100.0)	89.5 (89.2-89.9)
No	10.9 (0.6-21.3)^c^	11.3 (6.4-16.1)	15.6 (15.2-16.0)	NA^d^	NA^d^	10.5 (10.1-10.8)
Mental health						
Lifetime diagnosed depression	5.7 (3.2-8.2)^b^	9.1 (4.4-13.8)	9.9 (9.6-10.2)	19.4 (2.0-36.7)^c^	18.1 (2.0-34.3)^c^	19.6 (19.2-20.0)
Frequent mental distress	10.6 (3.7-17.5)^c^	9.0 (4.2-13.8)	8.0 (7.7-8.2)	4.7 (0.3-9.0)^b^^,^^c^	NA^d^	11.5 (11.2-11.9)
Extreme distress	NA^d^	5.3 (1.0-9.5)^c^	3.4 (3.3-3.6)	NA^d^	NA^d^	4.8 (4.6-5.0)
Mean mentally unhealthy days	3.3 (1.2-5.3)^c^	2.8 (1.5-4.0)	2.7 (2.6-2.8)	2.3 (0.9-3.8)^c^	5.5 (0.7-10.2)^c^	3.9 (3.8-4.0)

Abbreviations: APR, adjusted prevalence ratio; GED, General Educational Development certification; NA, not applicable.

^a^ States contributing data were: Alaska, California, Colorado, Connecticut, Delaware, Florida, Georgia, Hawaii, Idaho, Illinois, Iowa, Kansas, Louisiana, Maryland, Massachusetts, Michigan, Minnesota, Mississippi, Missouri, Montana, Nebraska, New Hampshire, New Jersey, New Mexico, New York, North Carolina, North Dakota, Pennsylvania, Rhode Island, South Carolina, Tennessee, Texas, Utah, Vermont, Washington, West Virginia, Wisconsin.

^b^ Significantly lower than all other industries.

^c^ Relative standard error 30% or higher; estimate is approximate and should be interpreted with caution.

^d^ Relative standard error 50% or higher; estimate is not reportable.

^e^ Significantly higher than all other industries.

^f^ Respondents selected other race or multiracial.

Most manual labor–intensive industries, including agriculture, forestry, fishing, and hunting; mining; utilities; construction; manufacturing; wholesale trade; and transportation and warehousing, had significantly lower unadjusted lifetime diagnosed depression prevalences (range: mining, 6.7% [95% CI, 3.9%-9.5%] to manufacturing, 12.2% [95% CI, 11.5%-13.0%]) than all workers combined (14.2% [95% CI, 13.9%-14.4%]) (Table 3). After adjustment, 4 of these industries had significantly lower lifetime diagnosed depression (APR range: mining, 0.60 [95% CI, 0.40-0.88] to construction, 0.79 [95% CI, 0.71-0.87]) than the reference group, public administration. Retail trade; educational services; health care and social assistance; arts, entertainment, and recreation; accommodation and food services; and other services, except public administration, had significantly higher unadjusted lifetime diagnosed depression prevalences (range: 15.6% [95% CI, 14.8%-16.3%] for educational services to 18.4% [95% CI, 17.3%-19.6%] for accommodation and food services) than all workers combined. After adjustment, 4 of these 6 industry groups were significantly higher for lifetime diagnosed depression (APR range: 1.13 [95% CI, 1.05-1.22] health care and social assistance to 1.15 [95% CI, 1.05-1.25] for retail trade) than public administration. Retail trade, management of companies and enterprises, and accommodation and food services had similarly higher unadjusted and adjusted FMD prevalences than the respective comparison groups. The groups with unadjusted extreme distress prevalences that were significantly higher than for all workers combined included retail trade (5.3% [95% CI, 4.8%-5.8%]) and accommodation and food services (6.8% [95% CI, 6.0%-7.7%]). OGE and mining had higher extreme distress prevalences (range: OGE, 5.7% [95% CI, 1.7%-9.7%] to mining, 6.3% [95% CI, 0.1%-12.4%]) than all workers combined, although the differences were not statistically significant.

**Table 3.** Workers’ Depression, Frequent Mental Distress, Extreme Distress, and Mentally Unhealthy Days by Industry, Behavioral Risk Factor Surveillance System, 37 States, 2015-2019^a^

NAICS code	Industry	Sample No, unweighted	Lifetime diagnosed depression		Frequent mental distress		Extreme distress, unadjusted, % (95% CI)	MUD by depression status, mean (95% CI)	
			Unadjusted, % (95% CI)	APR (95% CI)^b^	Unadjusted, % (95% CI)	APR (95% CI)^b^		Yes	No
All workers combined		498 351	14.2 (13.9-14.4)	NA	9.6 (9.4-9.8)	NA	4.1 (3.9-4.2)	9.5 (9.4-9.7)	2.2 (2.2-2.3)
11	Agriculture, forestry, fishing, and hunting	19 165	9.3 (7.2-11.3)^c^	0.76 (0.61-0.94)	7.2 (5.0-9.4)	0.86 (0.62-1.18)	3.5 (1.8-5.2)	8.3 (6.0-10.5)	1.8 (1.2-2.3)
212	Mining	969	6.7 (3.9-9.5)^c^	0.60 (0.40-0.88)	10.2 (3.8-16.5)^d^	1.38 (0.75-2.53)	6.3 (0.1-12.4)^d^	9.8 (5.7-13.8)	2.7 (0.7-4.7)^d^
211, 213	Oil and gas extraction	2718	10.0 (5.5-14.6)	0.88 (0.57-1.38)	10.0 (5.3-14.7)	1.26 (0.79-2.02)	5.7 (1.7-9.7)^d^	12.7 (5.7-19.8)	1.9 (1.2-2.6)
22	Utilities	5296	8.2 (6.6-9.8)^c^	0.72 (0.59-0.87)	7.3 (5.5-9.0)	1.02 (0.78-1.32)	3.7 (2.3-5.0)	10.7 (8.7-12.8)	1.8 (1.4-2.3)
23	Construction	36 051	8.9 (8.2-9.5)^c^	0.79 (0.71-0.87)	8.5 (7.8-9.3)	1.05 (0.92-1.20)	4.1 (3.5-4.6)	9.3 (8.6-10.1)	2.2 (2.0-2.4)
31, 33	Manufacturing	44 063	12.2 (11.5-13.0)^c^	0.96 (0.88-1.05)	8.6 (7.9-9.3)	1.03 (0.91-1.17)	3.7 (3.2-4.1)	9.4 (8.7-10.2)	2.0 (1.8-2.1)^c^
42	Wholesale trade	6186	10.9 (9.0-12.7)^c^	0.86 (0.73-1.02)	7.5 (6.1-9.0)	0.92 (0.75-1.14)	2.9 (2.1-3.7)^c^	8.8 (7.0-10.7)	1.7 (1.5-2.0)^c^
44, 45	Retail trade	42 065	17.7 (16.8-18.7)	1.15 (1.05-1.25)	12.5 (11.7-13.3)^e^	1.23 (1.10-1.39)	5.3 (4.8-5.8)^e^	11.0 (10.3-11.7)^e^	2.5 (2.3-2.7)
48, 49	Transportation and warehousing	19 374	11.2 (10.2-12.2)	0.95 (0.85-1.06)	9.1 (8.2-10.0)	1.07 (0.93-1.23)	4.1 (3.4-4.7)	10.2 (9.1-11.2)	2.1 (1.9-2.3)
51	Information	8727	15.1 (13.4-16.9)	1.15 (1.00-1.31)	10.0 (8.1-11.9)	1.24 (1.01-1.53)	4.1 (3.1-5.0)	9.6 (8.2-11.0)	2.4 (2.0-2.8)
52	Finance and insurance	20 786	13.9 (12.8-15.1)	0.96 (0.87-1.06)	8.2 (7.2-9.2)	1.04 (0.89-1.22)	3.1 (2.6-4.0)	8.2 (7.3-9.1)^c^	2.1 (1.8-2.3)
53	Real estate and rental and leasing	10 222	12.3 (10.9-13.7)	0.88 (0.78-1.00)	9.0 (7.7-10.3)	1.12 (0.94-1.34)	3.3 (2.6-4.0)	9.1 (7.8-10.3)	2.1 (1.8-2.4)
54	Professional, scientific, and technical services	27 670	14.0 (13.0-15.1)	1.06 (0.96-1.17)	8.1 (7.2-9.0)	1.12 (0.97-1.29)	3.3 (2.7-3.8)^c^	8.6 (7.9-9.3)^c^	2.0 (1.8-2.2)
55	Management of companies and enterprises	687	12.5 (4.5-20.5)^d^	0.89 (0.48-1.65)	16.5 (6.0-27.1)^d^	2.03 (1.13-3.65)	4.4 (0.8-8.1)^d^	10.2 (5.5-14.8)	3.4 (1.4-5.4)
56	Administrative and support and waste management services	13 597	13.8 (12.5-15.1)	1.00 (0.89-1.13)	11.0 (9.8-12.2)	1.17 (1.01-1.36)	5.0 (4.2-5.8)	11.0 (9.7-12.2)	2.2 (1.9-2.4)
61	Educational services	53 375	15.6 (14.8-16.3)^e^	1.04 (0.96-1.13)	7.8 (7.2-8.5)	1.01 (0.89-1.14)	2.9 (2.5-3.3)^c^	8.1 (7.5-8.6)^c^	2.1 (1.9-2.2)
62	Health care and social assistance	81 932	18.2 (17.5-18.9)^e^	1.13 (1.05-1.22)	9.8 (9.3-10.3)	1.06 (0.95-1.18)	4.3 (4.0-4.7)	8.9 (8.4-9.3)^c^	2.2 (2.1-2.3)
71	Arts, entertainment, and recreation	8210	17.2 (15.0-19.5)^e^	1.15 (0.99-1.33)	11.0 (9.2-12.9)	1.17 (0.97-1.41)	4.6 (3.2-6.1)	10.6 (9.2-12.0)	2.4 (2.0-2.8)
72	Accommodation and food services	22 895	18.4 (17.3-19.6)^e^	1.13 (1.03-1.25)	14.8 (13.7-15.8)^e^	1.27 (1.12-1.44)	6.8 (6.0-7.7)^e^	11.7 (10.9-12.5)^e^	3.2 (3.0-3.5)^e^
81	Other services (except public administration)	23 841	16.3 (15.2-17.4)^e^	1.11 (1.01-1.23)	10.3 (9.3-11.2)	1.10 (0.96-1.26)	4.2 (3.6-4.8)	9.6 (8.8-10.4)	2.3 (2.1-2.5)
92	Public administration	30 887	13.2 (12.3-14.1)	1 [Reference]	7.7 (6.9-8.4)	1 [Reference[	2.9 (2.4-3.3)^c^	8.5 (7.8-9.2)^c^	1.8 (1.7-2.0)

Abbreviations: APR, adjusted prevalence ratio; MUD, mean mentally unhealthy days; NA, not applicable; NAICS, North American Industrial Classification System.

^a^ States contributing data were: Alaska, California, Colorado, Connecticut, Delaware, Florida, Georgia, Hawaii, Idaho, Illinois, Iowa, Kansas, Louisiana, Maryland, Massachusetts, Michigan, Minnesota, Mississippi, Missouri, Montana, Nebraska, New Hampshire, New Jersey, New Mexico, New York, North Carolina, North Dakota, Pennsylvania, Rhode Island, South Carolina, Tennessee, Texas, Utah, Vermont, Washington, West Virginia, Wisconsin.

^b^ Adjusted prevalence ratio; for age, sex, race/ethnicity, education level, and marital/coupled status.

^c^ Significantly lower than all workers combined.

^d^ Relative standard error ≥30%; estimate is approximate and should be interpreted with caution.

^e^ Significantly higher than all workers combined.

Management; computer and mathematical; architecture and engineering; protective service; farming, fishing, and forestry; construction workers and supervisors; extraction; installation, maintenance, and repair; production; and transportation and material moving had significantly lower unadjusted lifetime diagnosed depression prevalences (range: 6.2% [95% CI, 2.7%-9.8%] for extraction to 10.3% [95% CI, 9.2%-11.3%] for installation, maintenance, and repair) than all workers combined (Table 4). After adjustment, only architecture and engineering, protective service, and construction were significantly lower for lifetime diagnosed depression than the reference group, transportation and material moving (APR range: 0.81 [95% CI, 0.72-0.91] for construction workers and supervisors to 0.85 [95% CI, 0.73-0.98] for architecture and engineering). Nine occupation groups, including community and social services; arts, design, entertainment, sports, and media; health care support; food preparation and serving related; and personal care and service had significantly higher unadjusted lifetime diagnosed depression prevalences (range: 16.1% [95% CI, 15.2%-17.0%] for education, training, and library to 20.5% [95% CI, 18.7%-22.4%] for community and social services) than all workers combined, and after adjustment, 8 of the 9, plus life, physical, and social science and legal were significantly higher than transportation and material moving (APR range: 1.13 [95% CI, 1.02-1.24] for office and administrative support to 1.47 [95% CI, 1.30-1.66] for community and social services). The prevalences of FMD in arts, design, entertainment, sports, and media; health care support; food preparation and serving; sales and related; and office and administrative support were significantly higher than the respective comparison groups in both unadjusted and adjusted analyses. The 5 occupation groups with significantly higher unadjusted extreme distress prevalences compared with all workers included health care support (6.6% [95% CI, 5.5%-7.8%]); food preparation and serving related (6.9% [95% CI, 5.9%-7.8%]); building and grounds cleaning and maintenance (5.2% [95% CI, 4.4%-6.0%]); personal care and service (5.8% [95% CI, 4.9%-6.8%]), and sales and related (4.8% [95% CI, 4.3%-5.3%]).

**Table 4.** Workers’ Depression, Frequent Mental Distress, Extreme Distress, and Mentally Unhealthy Days by Occupation, Behavioral Risk Factor Surveillance System, 37 States, 2015-2019^a^

SOC code	Occupation group	Sample No. unweighted	Lifetime diagnosed depression		Frequent mental distress		Extreme distress, unadjusted, % (95% CI)	MUD by Depression status, mean (95% CI)	
			Unadjusted, % (95% CI)	APR (95% CI)^b^	Unadjusted, % (95% CI)	APR (95% CI)^b^		Yes	No
All workers combined		461 829	14.2 (13.9-14.4)	NA	9.6 (9.4-9.8)	NA	4.1 (3.9-4.2)	9.5 (9.4-9.7)	2.2 (2.2-2.3)
11-0000	Management (occupations)	56 882	11.7 (10.9-12.4)^c^	0.91 (0.82-1.0)	7.4 (6.8-7.9)^c^	0.93 (0.82-1.05)	3.2 (2.8-3.6)^c^	8.1 (7.4-8.8)	1.9 (1.8-2.1)
13-0000	Business and financial	20 412	14.6 (13.3-15.9)	1.05 (0.94-1.19)	7.4 (6.4-8.3)^c^	0.89 (0.76-1.05)	2.8 (2.1-3.4)^c^	8.6 (7.5-9.6)	1.7 (1.6-1.9)
15-0000	Computer and mathematical	13 357	12.0 (11.0-13.1)^c^	1.09 (0.97-1.22)	7.1 (5.9-8.2)^c^	0.98 (0.81-1.18)	2.7 (2.1-3.2)^c^	8.3 (7.4-9.2)	1.8 (1.6-2.1)
17-0000	Architecture and engineering	12 537	9.0 (7.9-10.1)^c^	0.85 (0.73-0.98)	6.0 (4.6-7.4)^c^	0.89 (0.69-1.15)	2.3 (1.6-3.0)^c^	6.9 (5.7-8.1)	1.8 (1.4-2.1)
19-0000	Life, physical, and social science	7010	15.1 (13.1-17.0)	1.20 (1.03-1.39)	5.5 (4.4-6.6)^c^	0.74 (0.60-0.93)	1.6 (1.1-2.0)^c^	6.8 (5.9-7.8)	1.7 (1.4-1.9)
21-0000	Community and social services	10 882	20.5 (18.7-22.4)^d^	1.47 (1.30-1.66)	9.0 (7.7-10.3)	1.06 (0.89-1.26)	2.7 (2.1-3.3)^c^	7.5 (6.7-8.3)	2.1 (1.9-2.4)
23-0000	Legal	6314	16.1 (13.4-18.8)	1.20 (1.01-1.42	9.0 (6.3-11.6)	1.22 (0.92-1.63)	3.7 (2.3-5.0)	8.6 (7.0-10.3)	2.1 (1.6-2.5)
25-0000	Education, training, and library	35 287	16.1 (15.2-17.0)^d^	1.07 (0.97-1.18)	7.9 (7.2-8.6)^c^	0.95 (0.82-1.08)	2.8 (2.4-3.2)^c^	7.5 (7.0-8.1)	2.2 (2.0-2.3)
27-0000	Arts, design, entertainment, sports, and media	10 006	18.6 (16.7-20.4)^d^	1.34 (1.17-1.52)	11.4 (9.4-13.3)	1.32 (1.09-1.60)	4.3 (2.9-5.6)	9.0 (7.9-10.1)	2.8 (2.3-3.3)
29-0000	Health care practitioners and technical	39 067	17.8 (16.8-18.9)^d^	1.16 (1.04-1.28)	8.2 (7.4-8.9)^c^	0.91 (0.79-1.04)	3.3 (2.8-3.8)^c^	7.6 (7.1-8.2)	2.0 (1.8-2.1)
31-0000	Health care support	10 872	20.9 (19.2-22.6)^d^	1.20 (1.06-1.34)	14.8 (13.3-16.3)^d^	1.19 (1.03-1.38)	6.6 (5.5-7.8)^d^	11.4 (10.3-12.5)	2.9 (2.6-3.2)
33-0000	Protective service	9197	9.8 (8.5-11.2)^c^	0.81 (0.70-0.95)	8.3 (7.0-9.6)	0.90 (0.75-1.08)	3.3 (2.4-4.1)	11.3 (9.7-12.9)	1.8 (1.6-2.0)
35-0000	Food preparation and serving related	15 166	20.1 (18.6-21.7)^d^	1.21 (1.09-1.36)	15.4 (14.0-16.7)^d^	1.20 (1.05-1.36)	6.9 (5.9-7.8)^d^	11.4 (10.5-12.3)	3.3 (2.9-3.6)
37-0000	Building and grounds cleaning and maintenance	17 599	13.8 (12.6-15.0)	1.03 (0.91-1.15)	10.7 (9.6-11.8)	1.03 (0.89-1.19)	5.2 (4.4-6.0)^d^	10.9 (9.9-12.0)	2.3 (2.0-2.5)
39-0000	Personal care and service	14 724	20.2 (18.6-21.8)^d^	1.21 (1.08-1.36)	13.0 (11.6-14.3)^d^	1.11 (0.96-1.28)	5.8 (4.9-6.8)^d^	10.8 (9.9-11.8)	2.5 (2.2-2.8)
41-0000	Sales and related	43 131	15.8 (15.0-16.6)^d^	1.06 (0.97-1.17)	11.7 (10.9-12.5)^d^	1.13 (1.01-1.27)	4.8 (4.3-5.3)^d^	10.9 (10.3-11.6)	2.5 (2.3-2.6)
43-0000	Office and administrative support	46 343	18.5 (17.5-19.4)^d^	1.13 (1.02-1.24)	11.1 (10.4-11.8)^d^	1.05 (0.93-1.18)	4.6 (4.1-5.0)	9.5 (8.9-10.1)	2.4 (2.2-2.5)
45-0000	Farming, fishing, and forestry	4629	9.8 (6.0-13.6)^c^	0.79 (0.55-1.15)	7.2 (3.7-10.7)	0.71 (0.44-1.14)	2.4 (1.5-3.2)^c^	9.2 (6.1-12.3)	1.5 (1.2-1.8)
47-XXXX	Construction workers and supervisors	26 909	8.7 (7.9-9.4)^c^	0.81 (0.72-0.91)	8.7 (7.8-9.6)	1.00 (0.87-1.14)	4.3 (3.6-5.0)	10.2 (9.1-11.3)	2.2 (2.0-2.5)
47-50XX	Extraction	1002	6.2 (2.7-9.8)^c^	0.57 (0.33-1.00)	6.7 (4.0-9.5)	0.78 (0.52-1.17)	2.9 (1.1-4.8)	8.3 (4.5-12.0)	1.8 (1.1-2.5)
49-0000	Installation, maintenance, and repair	15 937	10.3 (9.2-11.3^c^)	0.90 (0.79-1.02)	10.2 (8.9-11.5)	1.15 (0.98-1.34)	4.8 (3.9-5.6)	11.0 (9.8-12.3)	2.4 (2.1-2.7)
51-0000	Production	20 638	12.9 (11.8-13.9)	1.01 (0.90-1.12)	9.4 (8.6-10.3)	0.98 (0.86-1.12)	4.2 (3.7-4.7)	9.5 (8.8-10.3)	2.0 (1.9-2.2)
53-0000	Transportation and material moving	23 928	11.5 (10.6-12.5)^c^	1 reference	9.4 (8.6-10.3)	1 reference	4.5 (3.9-5.2)	10.5 (9.5-11.5)	2.1 (1.9-2.3)

Abbreviations: APR, adjusted prevalence ratio; MUD, mean mentally unhealthy days; NA, not applicable; SOC, US Standard Occupation Classification.

^a^ States contributing data were: Alaska, California, Colorado, Connecticut, Delaware, Florida, Georgia, Hawaii, Idaho, Illinois, Iowa, Kansas, Louisiana, Maryland, Massachusetts, Michigan, Minnesota, Mississippi, Missouri, Montana, Nebraska, New Hampshire, New Jersey, New Mexico, New York, North Carolina, North Dakota, Pennsylvania, Rhode Island, South Carolina, Tennessee, Texas, Utah, Vermont, Washington, West Virginia, Wisconsin.

^b^ Adjusted prevalence ratio; for age, sex, race and ethnicity, education level, and marital/coupled status.

^c^ Significantly lower than all workers combined.

^d^ Significantly higher than all workers combined.

## Discussion

Among all workers combined, sex, age, race and ethnicity, educational attainment, and no health care coverage were associated with 1 or more poor mental health outcomes. Lifetime diagnosed depression prevalence in female workers was double that for male workers, consistent with previous studies.^34^ Female and younger workers, as well as those not in a couple, had higher prevalences for each of the outcomes of lifetime diagnosed depression, FMD, and extreme distress. Being non-Hispanic White was associated with a higher prevalence of lifetime diagnosed depression but not the other outcomes. Extreme distress appears to be inversely associated with age and educational attainment and was higher in jobs with lower educational requirements. Across all sociodemographic groups, the mean MUD are 3 to 4 times higher among workers who reported lifetime diagnosed depression. The association of mean MUD with lifetime diagnosed depression is consistent with the validity of the MUD survey question, which is used to calculate FMD and extreme distress. It is notable that workers with no health care coverage had a significantly higher prevalences of FMD, extreme distress, and mean MUD, but not lifetime diagnosed depression, which requires diagnosis by a licensed health care professional, suggesting the impact of having no health insurance.

After controlling for the above sociodemographic factors, many of the significant differences in the mental health outcomes among industry and occupation groups remained, suggesting that work-related factors are important. When comparing worker groups, it is important to consider all mental health outcomes and interpret the results with caution. For example, mining, OGE, and construction industries and the equivalent extraction and construction occupation groups had a significantly lower adjusted prevalences of lifetime diagnosed depression than the reference groups. Paradoxically, these industries have the highest male suicide rates among all US industries.^9,10^ Low lifetime diagnosed depression prevalences and high suicide rates in predominately male industries and occupations might in part be due to reduced care-seeking for mental health problems among male workers stemming from unmeasured factors such as stigma or absence of health care services near rural or remote work sites.^35,36^ It is notable that the industries and occupations with the highest prevalences of depression, FMD, and extreme distress are diverse, including groups that do manual labor work, and those that do professional work.^37^

### Limitations

Our study had several limitations. BRFSS is a general population survey, and the number of respondents in the small industry and occupation groups results in lower precision or nonreportable prevalence estimates. Misclassification of industry and occupation is possible because of errors or ambiguity in self-reporting. The BRFSS depression question includes minor depression, which is not a depressive disorder in the *Diagnostic and Statistical Manual of Mental Disorders (Fifth Edition)*.^38^ The association of MUD with clinical diagnoses is unknown. The temporal relationship between lifetime depression diagnosis and exposure to an industry or occupation is unknown, and causal inferences are not possible. Responses in BRFSS may be affected by recall and social desirability biases. Results may not be representative of all states. BRFSS does not contain questions regarding work psychosocial hazards or stress. Using nonoverlapping CIs to define statistically significant differences between groups’ unadjusted estimates is conservative and may fail to identify significant (*P* < .05) associations.

## Conclusions

In this cross-sectional study, the prevalences of lifetime diagnosed depression, FMD, extreme distress, and of mean MUD varied significantly among workers by sociodemographic factors, industry, and occupation. Some of the observed differences may have been due to unmeasured factors, such as existing chronic illnesses. Workplaces can play a role in identifying and reducing psychosocial hazards and promoting workers’ mental health. Psychosocial intervention studies are promising, but the evidence for effectiveness is modest, with low certainty.^37,39^ More research is needed to evaluate work-related factors and workplace intervention effectiveness.^40^ NIOSH Total Worker Health offers integrated approaches to assist employers in improving workers’ mental and physical health.^40^

## Notes

Supplement.
Data Sharing Statement
