# Supplementary material for: US Workers’ Self-Reported Mental Health Outcomes by Industry and Occupation
Source: JAMA Netw Open. 2025 Jun 6;8(6):e2514212. doi: 10.1001/jamanetworkopen.2025.14212 (PMC12144615; doi:10.1001/jamanetworkopen.2025.14212)
Supplement: Supplement. — Data Sharing Statement [file jamanetwopen-e2514212-s001.pdf]

Data Sharing Statement

Sussell. US Workers’ Self-Reported Mental Health Outcomes by Industry and Occupation. *JAMA Netw Open*. Published June 06, 2025. doi:10.1001/jamanetworkopen.2025.14212

Data
Data available: No

Additional Information
Explanation for why data not available: CDC classifies BRFSS datasets that include participants' State of residence and their specific industry and occupation sensitive data, due to the risk of inferential disclosure of identity. This would particularly the case for persons in uncommon industries or occupations in a State. Limited access to the data are available by submitting a research proposal to the CDC Research Data Centers.
